# Naturally-occurring serotype 3 *Streptococcus pneumoniae* strains that lack functional pneumolysin and autolysin have attenuated virulence but induce localized protective immune responses

**DOI:** 10.1371/journal.pone.0282843

**Published:** 2023-03-10

**Authors:** Ηαnnah E. Wong, Panagiotis Tourlomousis, Gavin K. Paterson, Steve Webster, Clare E. Bryant

**Affiliations:** 1 Department of Veterinary Medicine, University of Cambridge, Cambridge, Cambridgeshire, United Kingdom; 2 Royal (Dick) School of Veterinary Studies and the Roslin Institute, University of Edinburgh, Easter Bush Campus, Midlothian, United Kingdom; Universidad de La Sabana, COLOMBIA

## Abstract

*Streptococcus pneumoniae* is an important cause of fatal pneumonia in humans. These bacteria express virulence factors, such as the toxins pneumolysin and autolysin, that drive host inflammatory responses. In this study we confirm loss of pneumolysin and autolysin function in a group of clonal pneumococci that have a chromosomal deletion resulting in a pneumolysin-autolysin fusion gene Δ(*lytA’-ply’*)593. The Δ(*lytA’-ply’*)593 pneumococci strains naturally occur in horses and infection is associated with mild clinical signs. Here we use immortalized and primary macrophage in vitro models, which include pattern recognition receptor knock-out cells, and a murine acute pneumonia model to show that a Δ(*lytA’-ply’*)593 strain induces cytokine production by cultured macrophages, however, unlike the serotype-matched *ply*^+^*lytA*^+^ strain, it induces less tumour necrosis factor α (TNFα) and no interleukin-1β production. The TNFα induced by the Δ(*lytA’-ply’*)593 strain requires MyD88 but, in contrast to the *ply*^+^*lytA*^+^ strain, is not reduced in cells lacking TLR2, 4 or 9. In comparison to the *ply*^+^*lytA*^+^ strain in a mouse model of acute pneumonia, infection with the Δ(*lytA’-ply’*)593 strain resulted in less severe lung pathology, comparable levels of interleukin-1α, but minimal release of other pro-inflammatory cytokines, including interferon-γ, interleukin-6 and TNFα. These results suggest a mechanism by which a naturally occurring Δ(*lytA’-ply’*)593 mutant strain of *S*. *pneumoniae* that resides in a non-human host has reduced inflammatory and invasive capacity compared to a human *S*. *pneumoniae* strain. These data probably explain the relatively mild clinical disease in response to *S*. *pneumoniae* infection seen in horses in comparison to humans.

## Introduction

*Streptococcus pneumoniae* is an important cause of infectious respiratory disease in humans. It causes a severe pneumonia characterised by the rapid development of respiratory failure [1]. Pneumococcal disease is a worldwide burden and is estimated to kill around one million children each year [1, 2]. The primary niche of *S*. *pnuemoniae* is the human nasopharynx and *S*. *pneumoniae* is an opportunistic pathogen of the lower respiratory tract [3]. Asymptomatic carriage is common and can reach a point prevalence of around 50%, mainly in children under 5 years old [4]. *S*. *pneumoniae* virulence factors include a polysaccharide capsule, the pore-forming cytolytic toxin pneumolysin (Ply) and the enzyme autolysin (LytA) which degrades the bacterial cell wall [5]. These factors induce an innate immune response, which contributes to the control of the infection, but concurrently facilitates bacterial invasion and host immunopathology.

Serotype 3 strains are heavily encapsulated [6, 7], and often have moderate but consistent human carriage rates with relatively low rates of invasive disease when compared to other serotypes [8, 9]. Specific examples include, UK data on 36 serotypes showing serotype 3 strains ranked equal 6^th^ with 5.35 acquisitions/100,000 child-years (range of all serotypes 0.36–32.10), and ranked 13th for invasive pneumococcal disease, with 0.40 cases/100,00 child-years (range of all serotypes 0–5.27) [8]. Despite the inclusion of serotype 3 within the 13-valent pneumococcal vaccine, the prevalence of serotype 3-related invasive and non-invasive disease remains relatively constant [10, 11]. Opsonophagocytic killing assays demonstrate low efficacy of PCV13 against serotype 3 strains [12], and analysis of global samples of the serotype 3 clonal complex-180 has suggested antibiotic use and non-vaccine antigens may currently be more important drivers of serotype 3 prevalence than vaccine usage [12, 13]. The persistence of serotype 3-related human disease is important because risk of death from pneumococcal pneumonia is associated with capsular serotype, and a meta-analysis of 16 serotypes showed that serotype 3 strains confer the second highest relative risk of death, approximately 1.9 times greater than the risk of death from the reference serotype 14, with only serotype 19F resulting in a higher risk [14].

In contrast to humans, *S*. *pneumoniae* infection in horses causes mild respiratory disease [15, 16]. The strains of *S*. *pneumoniae* isolated from horses that have been genetically analysed to date are all capsular serotype 3 and have a 7 kb chromosomal deletion that includes the 3’ region of autolysin and the 5’ region of pneumolysin creating a non-functional *lytA-ply* fusion gene [17]. The inflammatory responses to equine pneumococcal strains carrying this gene deletions have not been defined. The clinical manifestation of *S*. *pneumoniae* infection in horses is lower respiratory tract inflammation, which variably involves a fever, cough, and ocular and nasal discharge [18–20]. Reports of horses with severe or fatal infections with *S*. *pneumoniae* are rare with only a single case report documenting a severe pneumococcal pneumonia in a foal [21].

Infection of mice with human strains of *S*. *pneumoniae* results in severe pneumonia mediated through pattern-recognition receptors (PRRs) [22, 23]. Nuclear Factor-κβ (NF-κβ) has been identified as a major hub of transcriptional responses to pneumococcal infection [24]. Several cytokines and PRRs, such as Tumour Necrosis Factor α (TNFα), Interleukin-1β (IL-1β), Toll-like receptor 2 (TLR2), Toll-like receptor 4 (TLR4), and NOD-, LRR- and pyrin domain-containing protein 3 (NLRP3) contribute to immunes response against *S*. *pneumoniae*. TNFα recruits neutrophils, reducing the incidence of bacteraemia in a mouse pneumonia model [25]. TLR4-deficient mice are more susceptible than wild type mice to fatal pneumococcal infection [26], while TLR2-deficient mice have a reduced ability to clear pneumococcal colonisation [27]. *IL*−*1β*^−/−^ mice demonstrate greater lung tissue damage and reduced survival in comparison to wild type mice in a pneumonia model [28]. NLRP3 activation reduces lung bacterial counts and increases survival in mouse pneumonia models [29, 30]. The intense inflammatory response, however, can cause host tissue damage progressing to acute respiratory distress syndrome [31]. Tissue damage is mediated by bacterial virulence factors, including pneumolysin and, indirectly, autolysin, combined with indirect bystander effects from the host inflammatory responses [32]. The less severe disease in horses infected with equine-associated *S*. *pneumoniae* is suggestive of an alternative pathogenesis to human-associated pneumococci expressing pneumolysin and autolysin.

Human-associated *S*. *pneumoniae* operates within a supragenome [33], which allows pneumococci to readily acquire characteristics to aid bacterial survival [34]. The recent expansion of the serotype 3 clonal complex-180 in response to antibiotic use demonstrates the difficulty in controlling *S*. *pneumoniae* with classical health policies [12, 13]. Equine-associated pneumococcal strains have relevance to the human disease, because understanding the characteristics of consistently low-pathogenic pneumococci may reveal factors that facilitate pneumococci to adopt low-pathogenic survival strategies. This information could ultimately better inform epidemiological predictions of the impact of new treatments or preventative health measures. In this study our aim was to define the inflammatory response and pathology induced by equine-associated pneumococcal strains and compare it to that induced by human-associated strains using immortalized and primary macrophage in vitro models, and a murine acute pneumonia model. Our hypothesis was that equine associated strains would result in attenuated host inflammatory responses and a reduction in cell death or tissue damage when compared to a *ply+lytA+* human strain.

## Materials and methods

### Bacterial strains

Eight strains of *S*. *pneumoniae*, A45, 2721, 409, 7573, A29, BLB1 No.5, BLB1 No.15, S/5528, all isolated from clinical equine cases, were obtained from Dr. Andrew Waller (Animal Health Trust, Newmarket, UK). The *ply*^+^ *lytA*^+^ serotype 3 human isolates, A66 [35] and strain 06–3499, were included for comparison, 06–3499 had been isolated from human invasive pneumococcal disease. A45 and A66 had publicly available genome sequences, accession number HE983624.1 and LN847353, respectively [36, 37]. The genomes of 2721, 409, 7573, A29, BLB1 No.5, BLB1 No.15, S/5528 were previously sequenced by Dr. Gavin Paterson (University of Edinburgh, UK), and Dr. Andrew Waller (Animal Health Trust, UK) using Illumina HiSeq 2000 technology and portions of the data were made available for use in this study.

### Preparation of *S*. *pneumoniae* for *in vitro* and *in vivo* infection

Bacteria were grown in 37°C static BHI broth (CM1135 Oxoid, Thermo Fisher Scientific, Basingstoke, Hampshire, UK)| until mid-log phase (0.6 OD_595_) and frozen in aliquots with glycerol. *S*. *pneumoniae* cultures were prepared from frozen glycerol stocks of known titre [38–40].

### Deoxycholate lysis

Bacteria were grown in 37°C static BHI broth until mid-log phase (0.5–0.6 OD_595_). Viable bacterial counts and optical density measurements were performed before and after addition of sodium deoxycholate (D6750-10G, Sigma Aldrich, Germany) [41].

### Semi-quantitative haemolysis assay

A bacterial pellet harvested from an active culture at 0.6 OD_595_ was resuspended in cold 1.5 ml 25 mM Tris, 50 mM NaCl pH7.5 with 1% bacterial protease inhibitor cocktail (P8465-5ML Sigma Aldrich, Germany) and sonicated (High Intensity Ultrasonic Processor, Sonics and Materials Inc, Vibra Cell, USA) as previously reported [42]. Lysate total protein was quantified using a bicinchoninic acid assay. Recombinant pneumolysin was obtained from Prof. Aras Kadioglu (University of Liverpool, UK) [29]. The semi-quantitative haemolysis assay was modified from previously reported methods [43, 44]. The bacterial lysate was serially diluted in PBS, with recombinant pneumolysin as a positive control. 5 mM Dithiothreitol (10197777001 Sigma Aldrich, Germany), final concentration, was added to each well. Equine erythrocytes (Defibrinated horse blood SR0050, Oxoid, Thermo Fisher Scientific, Basingstoke, Hampshire, UK) washed in PBS were added to all wells resulting in 0.7% (v/v). The plate was covered and incubated at 37°C for 30 min and then at 21°C for 30 min before immediate visual assessment. The end point was 50% lysis. Haemolytic titre is the reciprocal of the dilution factor.

### Immortalised murine bone marrow derived macrophages

Immortalised murine bone marrow-derived macrophages (iBMDM) were a gift from Prof. Douglas Golenbock (University of Massachusetts, USA). [45] iBMDM were cultured in Dulbecco’s Modified Eagle’s medium (DMEM) (D6046, Sigma Aldrich, Germany) supplemented with 10% Fetal Calf Serum (FCS) (SH3007003 Cytiva Hyclone, Thermo Fisher Scientific, Basingstoke, Hampshire, UK), 2 mM L-glutamine (59202C, Sigma Aldrich, Germany), 100 U/ml penicillin and 100 μg/ml streptomycin (Penicillin-Streptomycin P4458, Sigma Aldrich, Germany). iBMDMs were seeded in 96 well plates at 1 x 10^6^ cells/ml unless otherwise stated.

### Animals

Mice were housed in a specific-pathogen free facility at the Department of Veterinary Medicine, University of Cambridge according to the Animal Scientific Procedures outlined by the UK Home Office regulations. Wild type C57Bl/6 mice were purchased from Charles River (C57BL/6NCrl Charles River, Harwell, UK). Tlr2^-/-^, Tlr4^-/-^, Tlr2/4^-/-^ on a C57BL/6 background were obtained from Shizuo Akira (Osaka University, Japan). Nlrp3^-/-^, Casp-1^-/-^(11^-/-^)  mice on a C57BL/6 background were produced by Millennium Pharmaceuticals and obtained from Kate Fitzgerald (University of Massachusetts, USA). All mouse colonies were bred independently. Mice were genotyped by PCR using standard protocols. Experiments were performed under and complied fully with Home Office guidelines. This research has been regulated under the Animals (Scientific Procedures) Act 1986 Amendment Regulations 2012 following ethical review by the University of Cambridge Animal Welfare and Ethical Review Body (AWERB). All work involving live animals complied with the University of Cambridge Ethics Committee regulations and was performed under the Home Office Project License number 80/2572.

### Isolation and culture of primary murine bone marrow-derived macrophages

Primary bone marrow-derived macrophages (BMDM) were prepared and cultured for 6–9 days as previously described [46]. Briefly, mice were killed by cervical dislocation and sterilised using 70% ethanol. The hind legs were removed at the hip joint and placed in chilled DMEM. The bone marrow flushed out and resuspended in complete BMM medium containing DMEM supplemented with 10% heat-inactivated FCS 5 mM L-glutamine and 20% L929 conditioned media. L929 conditioned media was prepared by growing L929 cells in RPMI 1640 (R4130 Sigma Aldrich, Germany) containing 10% FCS and 2 mM L-glutamine to confluency for 2 weeks. Supernatant was collected and filter-sterilised using 0.22 μM filters (SLGP033RB Millipore, Watford, UK). Bone marrow cells were seeded on to sterile Petri dishes (9cm diameter, 10 mL per dish) and grown for 3 days at 37°C 5% CO_2_, upon which, 10 mL of fresh BMM media were added and cells grown for a further 3 days before use. Primary BMDMs were seeded in 96 well plates at 1 x 10^6^ cells/mL unless otherwise stated.

### Acute pneumonia model with *S*. *pneumoniae*

Mice were lightly anaesthetised using 3–4% isofluorane and 50 μL of inoculum was pipetted between both nostrils. Control mice received PBS only. Mice were euthanased by asphyxiation in CO_2_ at 6h, 24 h and 36 h after pneumococcal infection. Blood collection was via cardiac puncture following euthanasia [47]. Bronchoalveolar lavage was performed by installing and re-aspirating 2 mL of sterile PBS via a tracheal cannula. Lungs were aseptically removed, washed in PBS and then homogenised in 5 mL of fresh PBS for 2 min using a Colworth Stomacher. Lung homogenates were 10-fold serially diluted in PBS, plated on blood agar base No.2 with horse blood (CM0271, Oxoid, Thermo Fisher Scientific, Basingstoke, Hampshire, UK) and incubated overnight at 37°C followed by enumeration of colony-forming units. Lung homogenate for cytokine analysis was diluted 1:1 with lysis buffer [48]. The 2x lysis buffer contained 300 mM NaCl, 2 mM CaCl_2_, 2mM MgCl_2_, 1% (v/v) Triton-X-100, pepstatin A, leupeptin, aprotinin (all at 20 ng/ml), pH7.4 (P8340, protease inhibitor cocktail, Sigma Aldrich, Germany). Lung tissue for histology were infused with 10% buffered formalin via tracheal catheterisation in situ, then collected whole, fixed for 48h in 10% buffered formalin, and embedded in paraffin. A single whole lung haematoxylin and eosin (H&E) stained-section was evaluated by a pathologist blinded to the groups. The scoring system was modified from a previously reported protocol [49]. Inflammation, necrosis and oedema were semi-quantitatively scored for airspaces (airways and alveolar spaces), interstitium and pleura. Lesion distribution was recorded using a scale of 0–4, zero representing no lesions and 4 representing diffuse pathology. Lesion severity was scored on a scale of 0–5 (zero representing no lesions, 5 representing marked extension into adjacent structures). Severity and distribution scores were multiplied to give a composite score for inflammation, necrosis and oedema respectively, and subsequently an overall total score. The presence of vascular fibrin thrombi scored an additional 10. Bacteria were visualised on Gram-stained sections, and recorded using a semiquantitative score modified from a previously reported protocol [50], the mean bacterial numbers from ten 928μm^2^ field of view were graded on a scale of 0–4 (zero representing no bacteria observed, score 1 <1–15 bacteria, score 2 15–50 bacteria, score 3 50–100 bacteria, and score 4 100+ bacteria).

### Cell viability assays

Cytotoxicity was quantified by measuring lactate dehydrogenase (LDH) activity using a modified protocol [46]. Uninfected cell cultures were used as standards. Following treatment, cells were washed three times in PBS and then lysed with 0.25% saponin at 37°C for 20 min. LDH activity was the measured using CytoTox96 Non-Radioactive Cytotoxicity Assay (G1780, Promega, Southampton, Hampshire, UK) according to manufacturer’s guidelines.

### Cytokine quantification

All cytokines were measured according to the manufacturer’s instructions. The OptEIA Mouse IL-1β set (559603, BD Biosciences, Wokingham, Berkshire, UK) and the TNFα DuoSet ELISA kit (DY410, R&D Systems, Abingdon, Oxfordshire, UK) were used to quantify cytokines secreted in cell culture supernatants. Serum and lung homogenates were screened by a custom flow cytometric multiplex bead array (Mouse Th1/Th2 10 plex kit FlowCytomix BMS820FF and Mouse Chemokine 6 plex kit FlowCytomix BMS821FF, Luminex Multiplex Assay, ProcartaPlex, ThermoFisher Scientific, Basingstoke, Hampshire, UK).

### Statistical analysis

Experiments involving cells were performed in triplicate. All experiments were repeated on different days; the number of separate experiments is stated in the figure legends. Statistical analysis of data was performed in GraphPad Prism 5 for Mac OS X (GraphPad Software Inc., La Jolla, CA) using two-tailed Mann-Whitney test, one-way ANOVA, or two-way ANOVA with Dunnett’s, Tukey’s or Sidak’s post-tests as specified in the figure legends. Statistical significance was designated when p < 0.05. Data are expressed as means and standard errors. P values are reported on the graphs.

## Results

### Pneumococcal strains isolated from horses contain a *lytA-ply* fusion gene, Δ(*lytA’-ply’*)593, and lack functional pneumolysin and autolysin

The presence of a 7 kb chromosomal deletion resulting in formation of *lytA-ply* fusion gene, Δ(*lytA’-ply’*)593, in strains of *S*. *pneumoniae* isolated from horses has been previously reported [17]. We investigated whether sequence data from eight pneumococcal strains isolated from clinical cases of respiratory disease or poor performance in horses contained the same genetic feature. The eight Δ(*lytA’-ply’*)593 strains were all sequence type ST6934 and serotype 3, and contained an identical Δ(*lytA’-ply’*)593 fusion gene (100% nucleotide identity), corresponding to bases 1–593 of *lytA* and bases 445–1416 of *ply* (S1 Fig). The fusion gene of these eight strains also contained an additional stop mutation, E50*, consistent with other published data [17]. Based on full length genomic BLAST, the closest related pneumococcal strain was the serotype 3 sequence type 387 strain A66, a *ply*^+^ *lytA*^+^ laboratory strain originally isolated from a human clinical case [35]. We have used A66 as a pneumolysin- and autolysin-expressing reference strain throughout this work.

The presence of a premature stop codon within the Δ(*lytA’-ply’*)593 gene suggested that translation of a functional product was unlikely. To confirm the lack of functional pneumolysin and autolysin, we investigated the haemolytic ability and bile solubility of the Δ(*lytA’-ply’*)593 strains. Pneumolysin lacks a standard secretion sequence [43], and the action of autolysin partially contributes to extracellular release of pneumolysin. We therefore used bacterial lysates instead of supernatants in this experiment to control for the lack of functional autolysin in the Δ(*lytA’-ply’*)593 strains. Serial dilutions of bacterial lysates mixed with equine red blood cells were visually assessed for haemolysis. Representative Δ(*lytA’-ply’*)593 strains, 2721 and 7573, were not haemolytic, whereas representative *ply*^+^ *lytA*^+^ strains, A66 and 06–3499 induced haemolysis (S2 Fig). We also measured sensitivity to deoxycholate-induced lysis through changes in culture optical density. A66 and 06–3499 cultures decreased optical density upon the addition of deoxycholate, whereas the optical density of cultures of Δ(*lytA’-ply’*)593 strains remained constant, resistant to lysis (S3 Fig). These data confirm that Δ(*lytA’-ply’*)593 pneumococci do not produce a functional pneumolysin or autolysin.

### Δ(*lytA’-ply’*)593 strains induce a transient peak of TNFα from BMDM and are unable to induce IL-1β

We then investigated the ability of Δ(*lytA’-ply’*)593 strains to instigate an inflammatory response *in vitro* and compared that to *ply*^+^*lytA*^+^ serotype 3 strains. Preliminary analysis established that all eight Δ(*lytA’-ply’*)593 strains induced TNFα production and macrophage cell death in wild type immortalised BMDM at a range of timepoints and MOIs, but that the strains were defective in IL-1β induction (S4 Fig). To investigate responses in primary macrophages, we then infected wild type primary mouse BMDMs with the human *ply*^+^*lytA*^+^ strain A66 and the Δ(*lytA’-ply’*)593 strain 2721, and measured TNFα and IL-1β production in cell supernatants and macrophage cell cytotoxicity.

**Fig 1:**
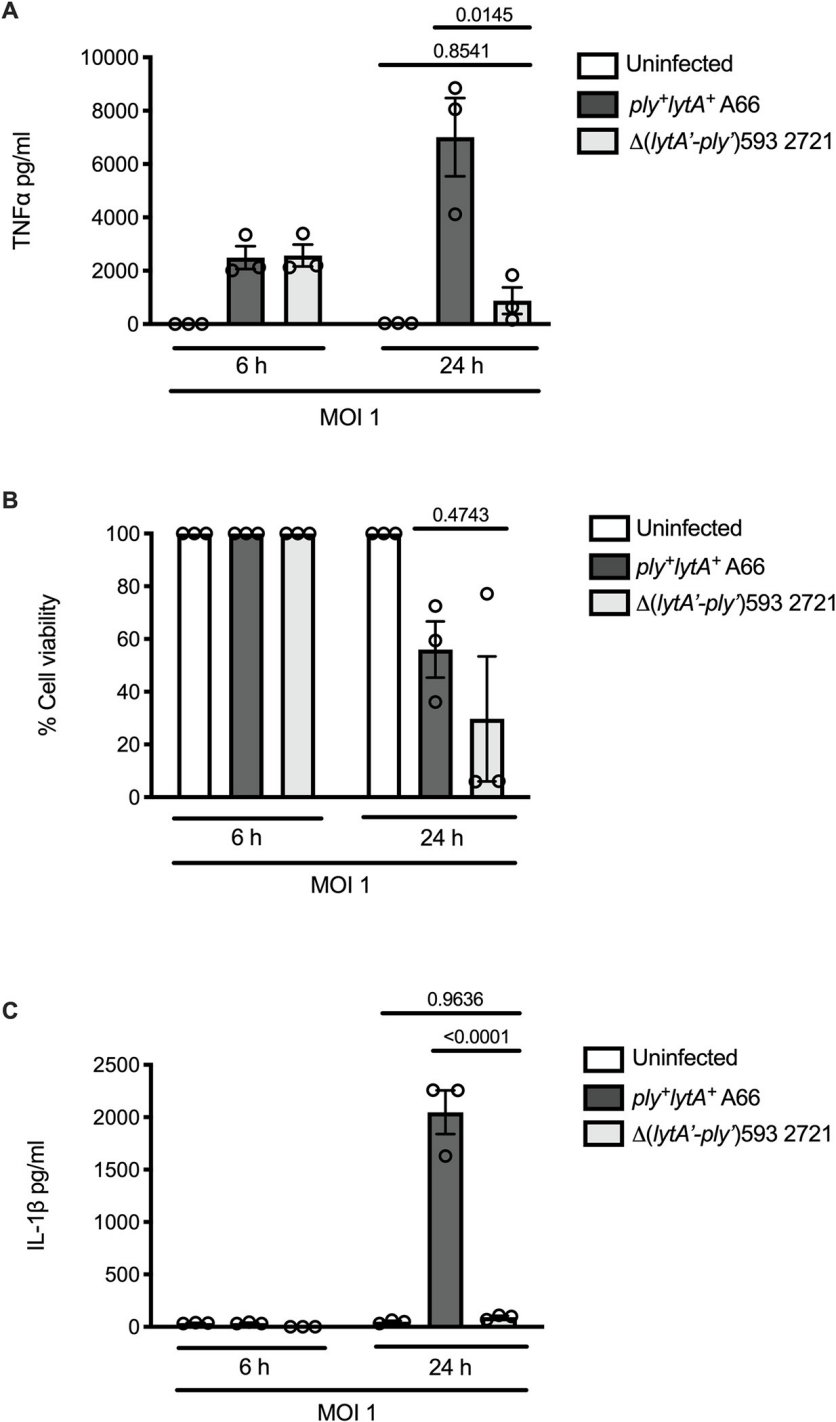
The Δ(*lytA’-ply’*)593 pneumococcal strain 2721 is less inflammatory than the *ply*^+^*lytA*^+^ serotype 3 strain A66 in murine primary BMDMs. Wild type primary BMDMs from C57Bl/6 mice were infected with *S*. *pneumoniae* Δ(*lytA’-ply’*)593 strain 2721 or *S*. *pneumoniae ply*^+^*lytA*^+^ strain A66 at an MOI 1 of for 6 h or 24 h. TNFα production in the supernatant was quantified by ELISA analysis (A). Cell viability was measured by LDH cytotoxicity assay (B). IL-1β production in the supernatant was quantified by ELISA (C). Data depicted represents the raw values, with the mean and SEM from three independent experiments. Statistical analysis: One-way ANOVA with Tukey’s multiple comparison test. Adjusted P-values reported.

**Fig 2:**
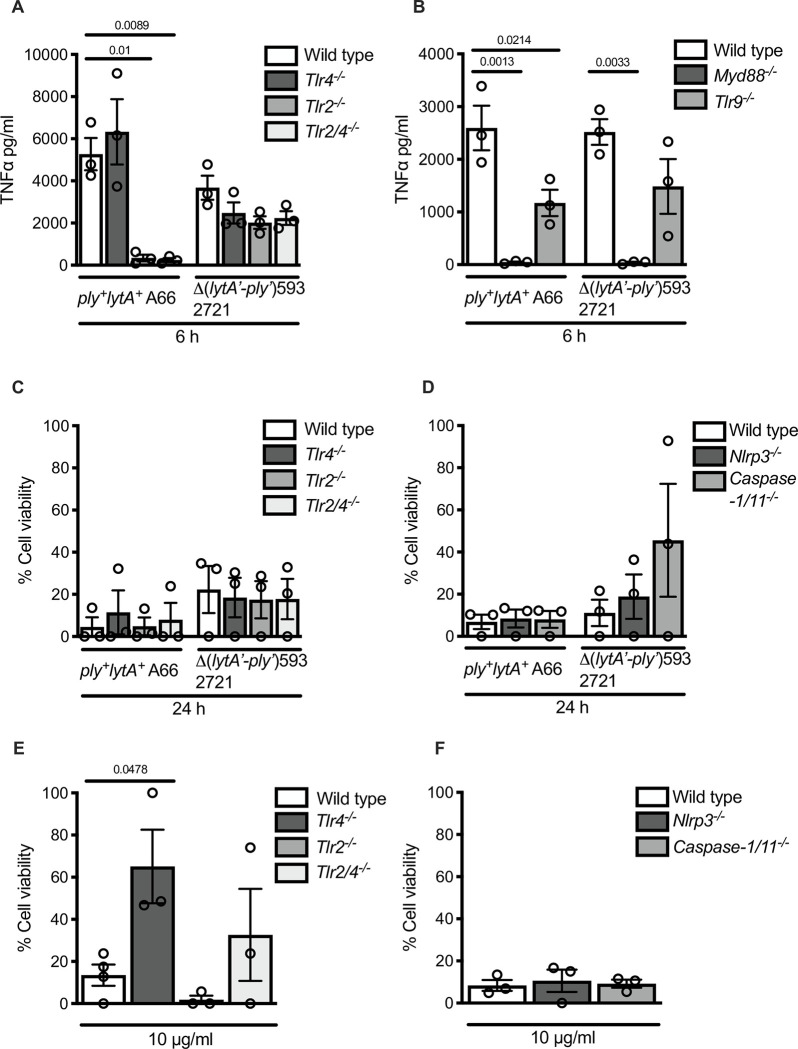
The macrophage inflammatory response to infection with Δ(*lytA’-ply’*)593 pneumococcal strain 2721 is MyD88-dependent, but has limited involvement of other PRR signalling pathways. Primary BMDMs from wild type, *Tlr4*^-/-^, *Tlr2*^-/-^ and *Tlr2/4*^-/-^ C57Bl/6 mice were infected with *ply*^+^*lytA*^+^ strain A66 or Δ(*lytA’-ply’*)593 strain 2721 at MOI 1 for 6 h. TNFα production in the supernatant was quantified by ELISA analysis (A). Immortalised BMMs from wild type, *Tlr9*^-/-^ and *Myd88*^-/-^ mice were infected with Δ(*lytA’-ply’*)593 strain 2721 and *ply*^+^*lytA*^+^ strain A66 at MOI 1 for 6 h (B). Primary BMDMs from wild type, *Tlr4*^−/−^, *Tlr2*^−/−^ and *Tlr2/4*^−/−^ mice were either infected with *ply*^+^*lytA*^+^ strain A66 or Δ(*lytA’-ply’*)593 strain 2721 at MOI 5 for 24 h (C) or stimulated with pneumolysin at 10 μg/ml for 24 h and cell viability was measured by the LDH cytotoxicity assay (E). Primary BMDMs from wild type, *Nlrp3*^*-/-*^, *Caspase 1*^−/−^*(Caspase 11*^−/−^*)* C57Bl/6 mice were either infected with Δ(*lytA’-ply’*)593 strain 2721 or *ply*^+^*lytA*^+^ strain A66 at MOI 10 for 24 h (D) or stimulated with purified pneumolysin at 10 μg/ml for 24 h (F) and cell viability was measured by the LDH cytotoxicity assay. Data depicted are the raw values, with the mean and SEM from three independent experiments. Statistical analysis: one-way ANOVA with Dunnet’s post-test, adjusted P-values reported.

In primary BMDM, the Δ(*lytA’-ply’*)593 strain 2721 and *ply*^+^*lytA*^+^ strain A66 both induced TNFα production at 6 h (Fig 1A). The *ply*^+^*lytA*^+^ strain A66 continued to induce substantial TNFα production at 24 h, however the Δ(*lytA’-ply’*)593 strain 2721 did not (Fig 2A). Both Δ(*lytA’-ply’*)593 strain 2721 and *ply*^+^*lytA*^+^ strain A66 induced macrophage cell death at 24 h, but not at 6 h, with no substantial difference between the two strains (Fig 1B). The *ply*^+^*lytA*^+^ strain A66 IL-1β induction was measurable by 24 h (Fig 1C), consistent with induction of IL-1β in response to inflammatory stimuli [51]. The Δ(*lytA’-ply’*)593 strain 2721 was unable to induce IL-1β production at the time points tested (Fig 1C).

### TNFα induction in response to Δ(*lytA’-ply’*)593 pneumococcus is MyD88-dependent but only partially dependent on TLR signalling

Our work so far shows that Δ(*lytA’-ply’*)593 strains of *S*. *pneumoniae* are able to induce TNFα and cell death, but fail to induce IL-1β in immortalised and primary mouse BMDMs. The inflammatory pathways involved in these responses are undefined. To assess whether MyD88, TLRs, NLRP3 and/or caspase-1/11 contributed to the TNFα production and cell death induced by Δ(*lytA’-ply’*)593 strains, we performed infection experiments using cells lacking these genes. Primary BMDMs from wild type, *tlr4*^-/-^, *tlr2*^-/-^ and *tlr2/4*^-/-^ mice were infected with Δ(*lytA’-ply’*)593 strain 2721 or *ply*^+^*lytA*^+^ strain A66. Infection with A66 resulted in TLR2-dependent TNFα release however, there was no significant impact of TLR4 on TNFα production (Fig 2A). Δ(*lytA’-ply’*)593-related TNFα induction was independent of TLR2 or TLR4 in primary BMDMs (Fig 2A). MyD88 is important for TNFα responses to *S*. *pneumoniae* as *MyD88*^*-/-*^ iBMDM infected for 6 h with Δ(*lytA’-ply’*)593 strain 2721 produced minimal TNFα (Fig 2B). To test the importance of TLR9 in the host response to Δ(*lytA’-ply’*)593 strains, we infected immortalised BMDM lacking *Tlr9* with Δ(*lytA’-ply’*)593 strain 2721 or *ply*^+^*lytA*^+^ strain A66. *Tlr9*^-/-^ cells infected with *ply*^+^*lytA*^+^ A66 produced reduced amounts of TNFα when compared to wild type cells, however TLR9 is not essential for TNFα induction in response to infection with the Δ(*lytA’-ply’*)593 strain (Fig 2B).

The contribution of specific PRRs and effector proteins to macrophage cell death induced by *S*. *pneumoniae* and purified pneumolysin were also investigated. TLR2, TLR4, NLRP3 and caspase-1/11 had no effect on cell death in primary BMDM induced by infection with Δ(*lytA’-ply’*)593 2721 or *ply*^+^*lytA*^+^ A66 (Fig 2C and 2D). TLR4, but not TLR2, NLRP3 or Caspase 1/11, appears to contribute to cell death induced by purified pneumolysin (Fig 2E and 2F).

### Infection of mice with Δ(*lytA’-ply’*)593 *S*. *pneumoniae* strain is associated with a neutrophilic lobar pneumonia, but it is asymptomatic with a limited pro-inflammatory cytokine response

**Fig 3:**
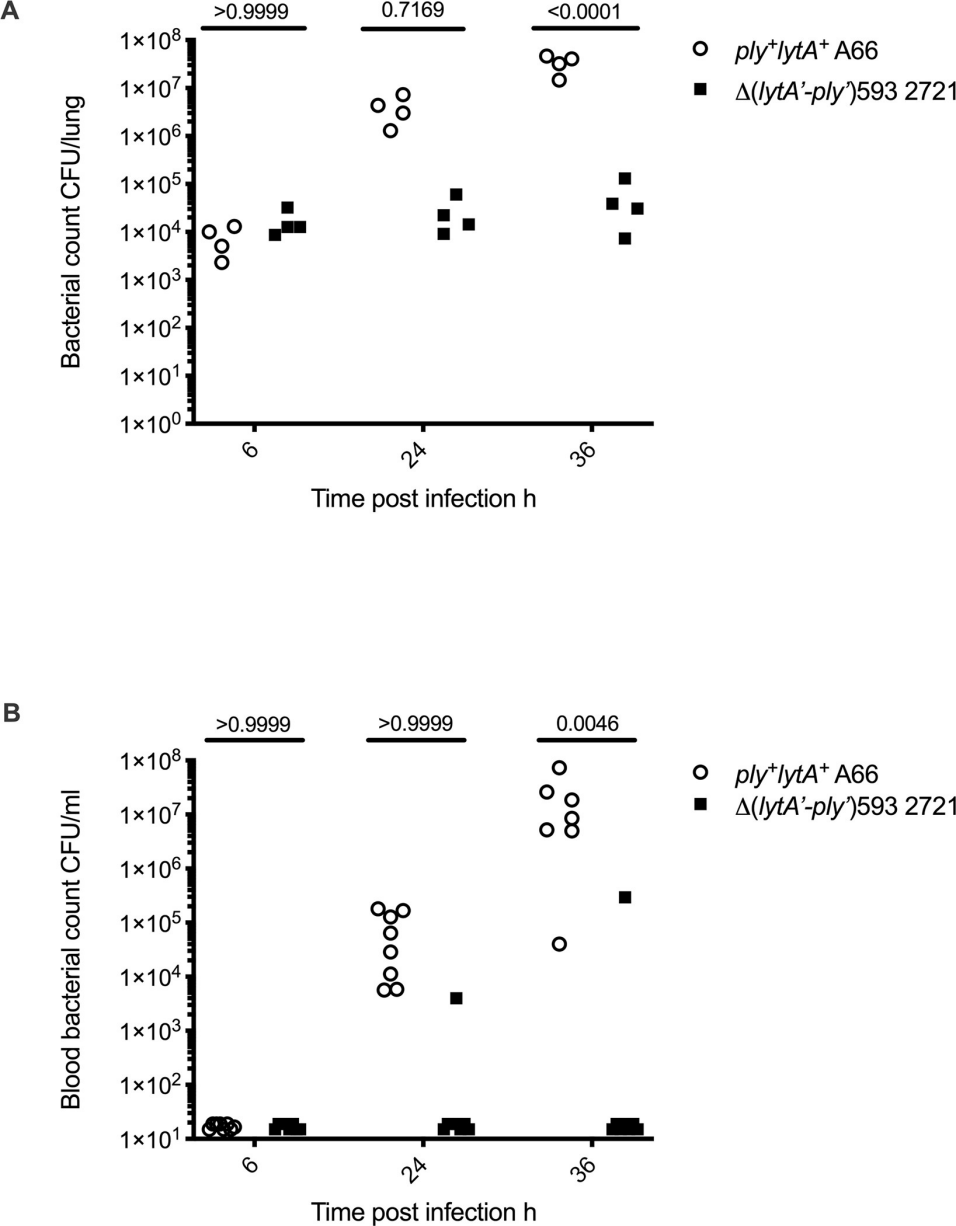
Δ(*lytA’-ply’*)593 strain 2721 persists in the lungs, but is unable to proliferate in this tissue or invade into the blood. Wild type C57Bl/6 mice were intranasally inoculated with 8 x 10^4^ CFU of *ply*^+^*lytA*^+^ strain A66 or 1.4 x 10^5^ CFU of Δ(*lytA’-ply’*)593 strain 2721 and bacterial counts were determined in lung homogenates (A). Each symbol represents one animal, data were generated from a single experiment. Wild type C57Bl/6 mice were intranasally inoculated with 3–8 x 10^4^ CFU of *ply*^+^*lytA*^+^ strain A66 or 8–14 x 10^4^ CFU of Δ(*lytA’-ply’*)593 strain 2721 and bacterial counts were determined in the blood (B). Each symbol represents one animal, 8 mice per group, data were pooled from two independent experiments. The lower limit of detection was 15 CFU/ml. Data analysed with two-way ANOVA followed by Sidak’s (A) or a Tukey’s (B) multiple comparison test. Adjusted P-values are reported.

The Δ(*lytA’-ply’*)593 strains induce a limited inflammatory response in macrophages *in vitro*, so we then investigated whether these differences were detectable in a mouse model of acute pneumonia. Wild type C57Bl/6 mice were intranasally inoculated with the *ply*^+^*lytA*^+^ strain A66 or the Δ(*lytA’-ply’*)593 strain 2721, and the bacterial burden was determined in the lungs and blood at 6, 24 and 36 h after infection. After 6 h of infection, both *ply*^+^*lytA*^+^ strain A66 and Δ(*lytA’-ply’*)593 2721 were present in similar numbers in the lungs but by 36 h post-infection the numbers of *ply*^+^*lytA*^+^ strain A66 had increased around 1000-fold whilst numbers of Δ(*lytA’-ply’*)593 strain 2721 remained unchanged (Fig 3A). This suggests that the Δ(*lytA’-ply’*)593 strain 2721 was able to persist within the lungs for the first 36 h following infection but was unable to proliferate. Mice infected with *ply*^+^*lytA*^+^ strain A66 developed clinical signs of generalised infection by 36 h post-infection, whereas mice infected with Δ(*lytA’-ply’*)593 strain 2721 did not demonstrate any signs of disease throughout the experiment. To investigate the capacity for systemic invasion, bacterial numbers of both strains were quantified in the blood (Fig 3B). The *ply*^+^*lytA*^+^ strain A66-innoculated mice were all bacteraemic by 24 h post-infection suggesting that *ply*^+^*lytA*^+^ strain A66 could invade from the lungs into the systemic circulation. Only two mice of the sixteen inoculated with Δ(*lytA’-ply’*)593 strain 2721 developed detectable bacteraemia, and neither demonstrated clinical signs of generalised infection.

**Fig 4:**
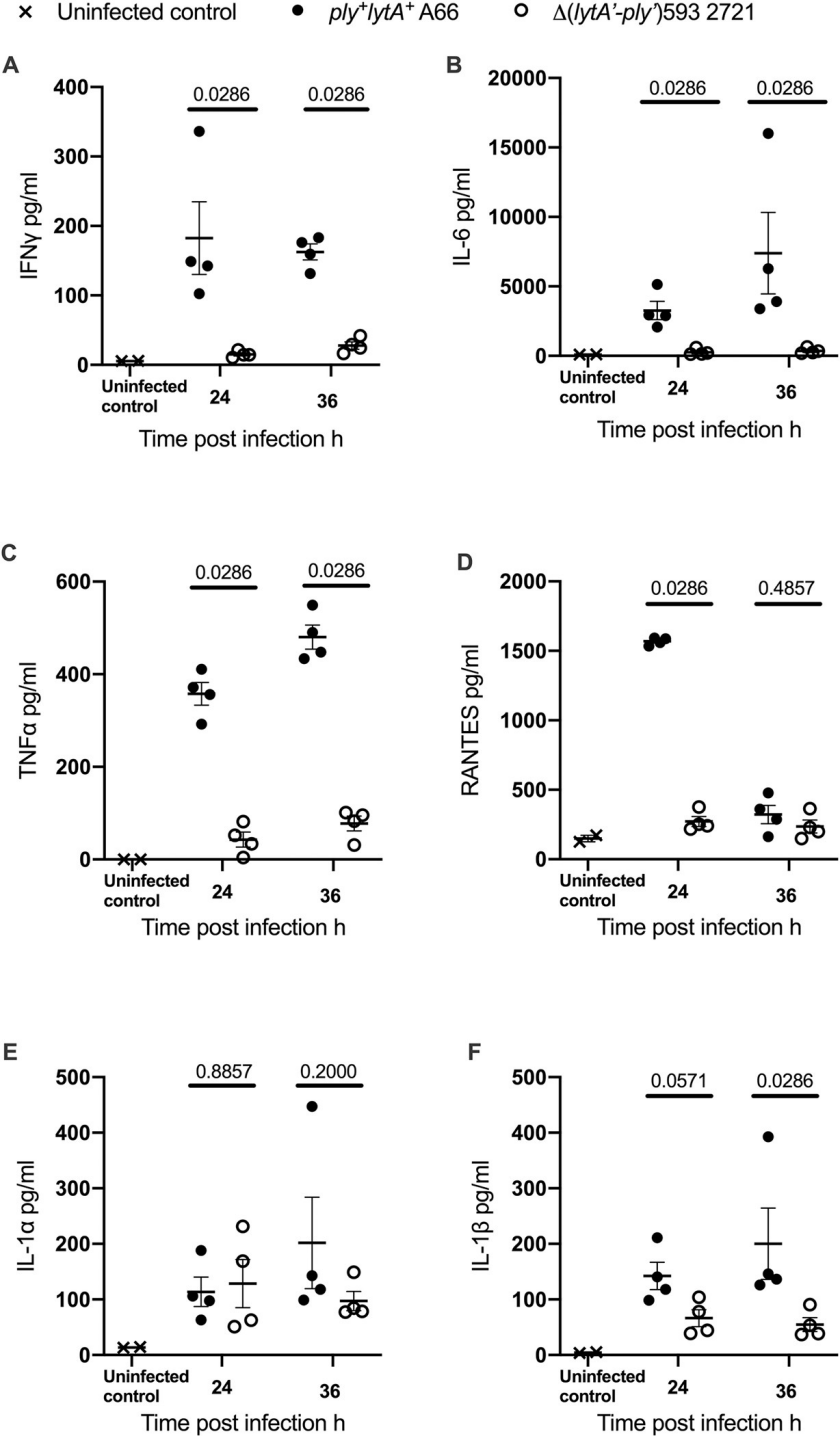
Δ(*lytA’-ply’*)593 strain, 2721, produces similar amounts of IL-1α in lung tissue to the *ply*^+^*lytA*^+^ strain A66 but induces less IFN-γ, IL-6, TNFα, RANTES, and IL-1β. Cytokines from lung homogenates of wild type C57Bl/6 mice intranasally inoculated with 8 x 10*4 CFU/mouse of *ply*^+^*lytA*^+^ strain A66 or 1.4 x 10*5 CFU-mouse of 2721 were measured by a Luminex bead array. Each data point represents one mouse and data were generated from a single experiment. The uninfected control group consists of mice inoculated intranasally with 50 μl PBS and humanely euthanased at 6 hours. Data analysed with a two-tailed Mann-Whitney test and P values reported.

The Δ(*lytA’-ply’*)593 strain 2721 induced similar levels of IL-1α production compared to the *ply*^+^*lytA*^+^ strain A66 in the lungs. Infection of mice with Δ(*lytA’-ply’*)593 strain 2721 induced much less IFNγ, TNFα, IL-1β or IL-6 in the lungs or serum (Fig 4A–4F and S5 Fig) compared to infection with *ply*^+^*lytA*^+^ strain A66. Infection with *ply*^+^*lytA*^+^ strain A66 induced detectable IFNγ, IL-6, TNFα, RANTES, IL-1α and IL-1β in the lungs of inoculated mice, and all but IL-1α in the bloodstream (Fig 4A–4F and S5 Fig).

**Fig 5:**
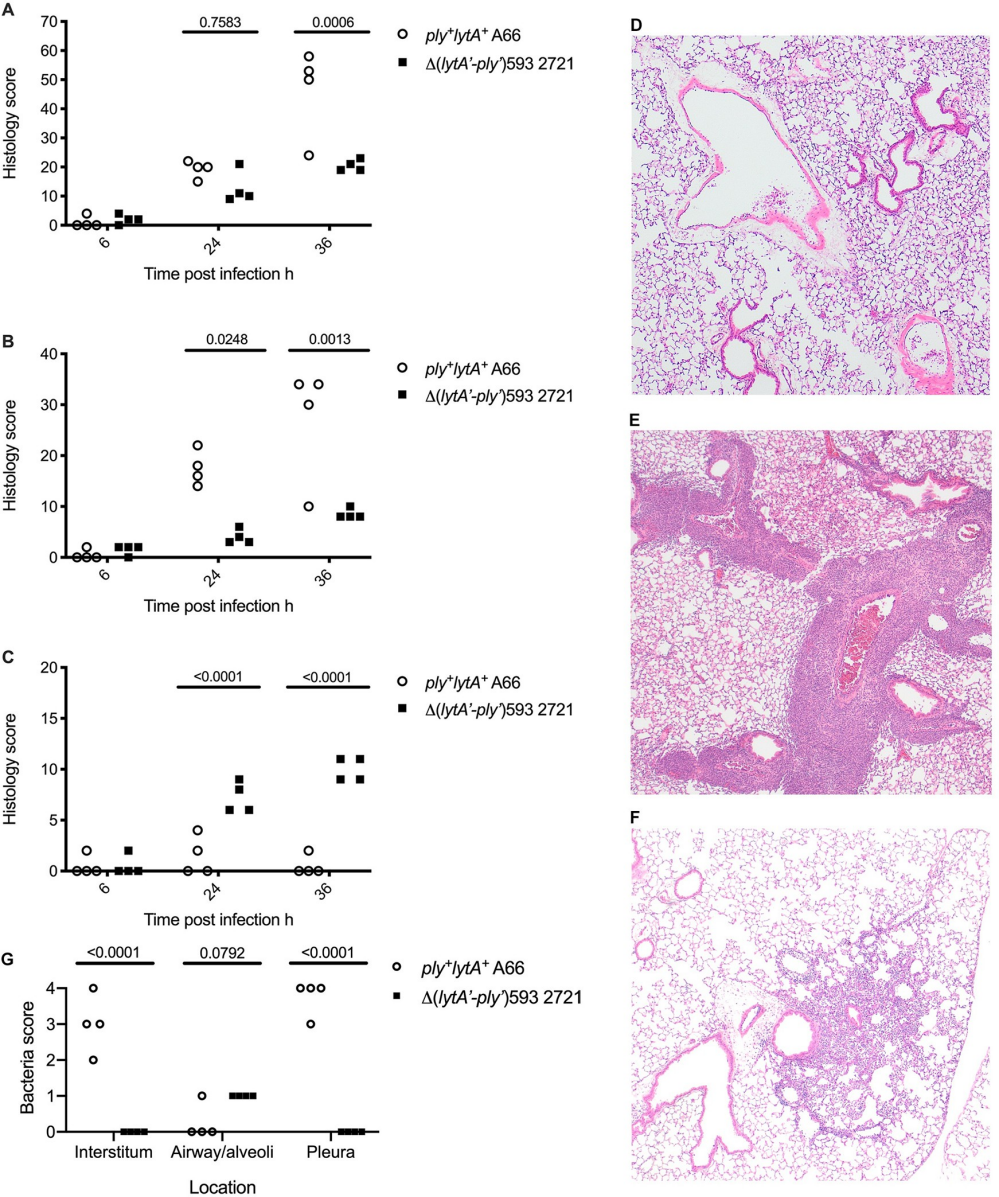
Infection of mice with the Δ(*lytA’-ply’*)593 strain, 2721, induces less severe and spatially distinct pathology to infection with *ply*^+^*lytA*^+^ strain, A66. Wild type C57Bl/6 mouse intranasally inoculated with 3 x 10^4^ CFU/mouse of A66 or 8 x 10^4^ 2721 CFU/mouse. Whole lungs were fixed and embedded in paraffin at the time points indicated, and haematoxylin and eosin (H&E)-stained sections assessed for pathology using a semi-quantitative histology score assessing inflammation, necrosis and oedema within the airways, interstitium and pleura. The overall histology score included all lung tissues (airways, interstitium and pleura) (A). The histology score of the interstitial tissues (B). The histology score of the air spaces (alveolar spaces/airways) (C). Representative micrograph of PBS-treated control, H&E stain magnification x20 (D). Representative micrograph of *ply*^+^*lytA*^+^ strain A66-induced pulmonary inflammation, H&E stain magnification x20, demonstrating marked interstitial inflammation (E). Representative micrograph of Δ(*lytA’-ply’*)593 strain 2721-induced pulmonary inflammation, H&E stain magnification x20, demonstrating localised intra-alveolar inflammation (F). Semiquantitative histology score representing mean bacterial numbers per 928μm^2^ (single x640 F22 field of view) within the interstitial tissues, alveolar spaces/airways and pleura (G). Data analysed with two-way ANOVA and a Tukey’s (A) or a Sidak’s (B,C,G) multiple comparison test. Adjusted P-values reported.

Pulmonary histology was used to investigate the differing *in vivo* profiles of Δ(*lytA’-ply’*)593 strain 2721 and *ply*^+^*lytA*^+^ strain A66. The *ply*^+^*lytA*^+^ strain A66-innoculated mice demonstrated higher inflammation scores at 36 h post-infection in comparison to mice inoculated with Δ(*lytA’-ply’*)593 strain 2721 (Fig 5A). The *ply*^+^*lytA*^+^ strain A66 induced inflammation within the interstitium, and this increased in severity over time (Fig 5B). As expected from previous studies [22, 47, 52], despite the high load of *ply*^+^*lytA*^+^ A66 in the lungs, histologically-detectable lesions within alveolar spaces were limited (Fig 5C).

PBS-treated control mice did not demonstrate lung pathology (Fig 5D), whereas inflammation induced by *ply*^*+*^*lytA*^*+*^ A66 strain was characterised by a marked neutrophilic peribronchiolar and perivascular infiltrate (Fig 5E). Inflammation induced by Δ(*lytA’-ply’*)593 strain 2721, on the contrary, was concentrated within the alveolar space, with limited interstitial and pleural involvement (Fig 5A–5C). The inflammation was predominantly neutrophilic resulting in a mild lobar pneumonia (Fig 5F). Gram-stained lung sections demonstrated that bacterial location corresponded with inflammatory foci. The majority of the *ply*^+^*lytA*^+^ strain A66, as recorded by a semiquantitative score representing bacterial numbers, were within the interstitium, whereas the Δ(*lytA’-ply’*)593 strain 2721 remained within alveolar spaces (Fig 5G). These data suggest that a major location of bacterial growth for *ply*^+^*lytA*^+^ strain A66 is the perivascular/bronchiolar interstitial tissue, whereas Δ(*lytA’-ply’*)593 strain 2721 remains predominantly within alveolar spaces.

## Discussion

Pneumococcal pneumonia is associated with a marked innate inflammatory response that is integral to infection control but can result in severe immunopathology associated with mortality. By investigating the innate inflammatory responses induced by a naturally-occurring *S*. *pneumoniae* mutant deficient in pneumolysin and autolysin, we sought to identify host-pathogen interactions that may contribute to reduced pneumococcal pathogenicity. We found that eight pneumococcal strains isolated from horses had an identical chromosomal deletion resulting in a non-functional Δ(*lytA’-ply’*)593 fusion gene. We demonstrated that Δ(*lytA’-ply’*)593 strains were less inflammatory and less invasive than a serotype-matched *ply*^+^*lytA*^+^ strain within the in vitro and in vivo models used here recapitulating the reported mild clinical presentation in horses [17]. Data from our *in vivo* model shows comparable clinical signs and pathology to those seen in an *in vivo* horse infection model with an equine-associated pneumococcal strain [20]. We propose that the loss of autolysin and pneumolysin, specifically in a serotype 3 strain, results in a pneumococcus deficient at both escaping the alveolus, and inducing pro-inflammatory mediators apart from IL-1α, and that these features contribute to the subclinical neutrophilic alveolar inflammation and control of bacterial numbers.

Functional pneumolysin is required for IL-1β production in mouse primary bone marrow macrophages, human peripheral blood mononuclear cells and THP-1 monocytes infected with *S*. *pneumoniae* [53]. The lack of IL-1β release from macrophages in response to Δ(*lytA’-ply’*)593 strains is consistent with that data, such that Δ(*lytA’-ply’*)593 strains, through failing to activate the inflammasome, cannot process this cytokine. The characteristics of the Δ(*lytA’-ply’*)593 strain *in vivo* has similarities with the reduced pneumococcal burdens and incidence of bacteraemia observed in *Nlrp3*^-/-^ or *Asc*^-/-^ mice [54]. The *Nlrp3*^-/-^ or *Asc*^-/-^ mice demonstrated a minimal neutrophilic response in the lung, however in contrast, the Δ(*lytA’-ply’*)593 strain induced intra-alveolar neutrophilic responses. The Δ(*lytA’-ply’*)593 strain also preserved IL-1α production despite other inflammatory cytokines remaining at baseline levels. Pneumococcal strains lacking pneumolysin induce more necrosis of alveolar macrophages compared to ply-containing bacteria [55], therefore phagocytosis of Δ(*lytA’-ply’*)593 bacteria could favour alveolar macrophage necrosis, with the resultant IL-1α release driving the neutrophil response [56, 57].

Pneumococcal strains expressing a non-cytolytic allele of pneumolysin, allele 5 [44, 58], still cause invasive disease [59], and the lack of inflammasome activation is suggested to facilitate entry to sterile areas [53, 60]. The majority of Δ(*lytA’-ply’*)593 bacteria remained within alveolar spaces, and we speculate that the concurrent lack of autolysin in Δ(*lytA’-ply’*)593 strains hinders invasion due to changes in capsule dynamics. Serotype 3 strains have a super mucoid capsule, produced by synthase-mediated synthesis in contrast to the wzy-mediated production of most other serotypes [61, 62]. Capsule production is continuous in serotype 3 strains and still occurs in in the absence of lytA [63–65]. The absence of lytA, however, does prevent capsule restructuring and hinders invasion across epithelial barriers [66, 67], therefore potentially contributing to the low-inflammatory, low-invasive, locally persistent phenotype observed in the Δ(*lytA’-ply’*)593 bacteria.

The TNFα response induced by Δ(*lytA’-ply’*)593 strains in vitro differed in PRR receptor dependency to serotype-matched *ply*^+^*lytA*^+^ strains. The Δ(*lytA’-ply’*)593 strain induced TNFα was solely dependent on MyD88 whilst the TNFα response to the *ply*^+^*lytA*^+^ strain was MyD88-dependent and variably dependent on TLR2, TLR4 and TLR9. These differences may be through differential TLR receptor redundancy or MyD88-dependent TLR-independent pathways such as IL-1R1. PAMP availability is expected to differ between Δ(*lytA’-ply’*)593 and *ply*^+^ *lytA*^+^ strains as the retention of a thick capsule hinders interaction with cell-surface PRRs [68]. TLR2 is activated by cell wall fragments [69], so the lack of autolysin in Δ(*lytA’-ply’*)593 strains could contribute to the loss of TLR2 dependency in the host response. Despite the differences, the MyD88-dependancy of both *ply*^*+*^*lytA*^*+*^ bacteria and Δ(*lytA’-ply’*)593-induced TNFα responses highlights that MyD88 is a critical signalling hub in the protective response to *S*. *pneumoniae*, as demonstrated by the recurrent and severe pneumococcal infections in young people with genetic MyD88 deficiency [70].

Host cell death is an important component of the inflammatory response and the cytotoxicity of the Δ(*lytA’-ply’*)593 strains suggests that cell death can be induced by pneumolysin- and autolysin- independent mechanisms in our *in vitro* model. We sought to define components involved in pneumococcal-induced host cell death but our data suggests that TLR2, TLR4, NLRP3, Caspase 1/11 were not important for the cytotoxicity of the Δ(*lytA’-ply’*)593 strain 2721 or the *ply*^+^*lytA*^+^ strain A66 in the models used here. One potential mechanism is death secondary oxidative stress from pneumococcal-derived hydrogen peroxide or reactive oxygen species [71–74], as an *in vitro* epithelial cell model, demonstrated pneumolysin-negative *S*. *pneumoniae* mutants retained cytotoxicity through hydrogen peroxide production [71]. The functional ability of the Δ(*lytA’-ply’*)593 strains to produce hydrogen peroxide has not yet been defined, however the Δ(*lytA’-ply’*)593 strain A45 (accession HE983624.1) has a *spxB* gene with 99.8% identity with *S*. *pneumoniae* strain Hu17 *spxB* (accession NZ-CP20549.1) suggesting that hydrogen peroxide production could be a feature of that strain [75]. The lack of effect of NLRP3 and Caspase 1/11 on host cell death during infections with *ply*^+^*lytA*^+^ strain A66 was unexpected, however receptor redundancy in macrophage death pathways may contribute to the lack of effect of the single knock-outs. TLR4, partially contributes to cell death induced by purified pneumolysin in our system. This is in contrast to *in vitro* data using C3H/HeJ splenocytes and dendritic cells, which demonstrated that TLR4 did not have a role in host cell death induced by purified pneumolysin [29]. Differences in pneumolysin concentration and host cell types may contribute to the observed differences in receptor involvement.

The incidence of *S*. *pneumoniae* isolated from non-human mammals is low and genetic analysis suggested that these isolates represented opportunistic infection by strains of human origin [76]. Rare animal-associated serotypes are identified, such as a serotype 19F strain with a unique sequence type found in guinea pigs across Europe [76]. The genetic similarity of *S*. *pneumoniae* strains isolated from horses could suggest a specific horse-associated lineage. There is no evidence to date of cross-species transmission of equine or human strains between affected horses and in-contact humans [15], and considering our data, this equine-associated genetic lineage may be unable to compete with fully virulent pneumococci.

The Δ(*lytA’-ply’*)593 strain 2721 was attenuated in its ability to induce Th1/Th2 related cytokines and chemokines in comparison to *ply*^+^*lytA*^+^ strain A66 from the lungs and blood of mice. It is not yet defined whether this reduction was secondary to differences in absolute bacterial numbers or due to intrinsic properties of the Δ(*lytA’-ply’*)593 strain. Further work varying the inoculum of the *in vivo* dose could be considered to further investigate the impact of bacterial numbers.

In this paper we demonstrate that naturally-occurring *S*. *pneumoniae* mutants deficient in pneumolysin and autolysin are less inflammatory and are less invasive than a serotype-matched *ply*^+^*lytA*^+^ strain. We propose that the dual lack of pneumolysin and autolysin within the specific capsule configuration of serotype 3 strain contributes to a subclinical IL-1α-associated neutrophilic inflammatory response. These findings further the understanding of pneumococcal host-pathogen interactions, and suggest further investigation of equine pneumococcal strains, including to determine whether the inflammatory responses are cross-protective against fully virulent serotype 3 strains.

## Supporting information

S1 Fig Schematic diagram of the equine *lytA-ply* fusion gene. (TIF)

S2 Fig Δ(*lytA’-ply’*)593 pneumococcal strains that lack pneumolysin are unable to induce lysis of equine erythrocytes. Using a semi-quantitative haemolysis assay, bacterial lysates were serially diluted in the presence of washed equine erythrocytes suspended in PBS and incubated, prior to visual assessment of the degree of erythrocyte lysis. Data depicted are individual data points with the median and range of three independent results, nd = not detected, limit of detection 20 HU/ml. *ply*^+^*lytA*^+^ strains: A66, 06–3499. Δ(*lytA’-ply’*)593 strains: 2721, 7573.
(TIF)

S3 Fig Liquid cultures of Δ(*lytA’-ply’*)593 pneumococci do not loose turbidity in the presence of deoxycholate, whilst cultures of *ply*^+^*lytA*^+^ pneumococci are rapidly lysed. Increasing concentrations of deoxycholate were added to liquid bacterial cultures and the optical density (OD_595_) of the culture measured after a 5 min incubation. Individual data points depicted of three independent experiments. *ply*^+^*lytA*^+^ strains: A66 and 06–3499 Δ(*lytA’-ply’*)593 strains: 2721 and 7573. 0.04% choline data analysed with one-way ANOVA and Dunnett’s multiple comparison post-test with adjusted P values reported. A66 compared to: 06–3499 p = 0.0877, 2721 p = 0.0015, 7573 p = 0.0033.
(TIF)

S4 Fig Δ(*lytA’-ply’*)593 strains of *S. pneumoniae* induce TNFα responses and cell death in murine iBMDM, but do not induce substantial IL-1β. Wild-type murine iBMDM were infected with Δ(*lytA’-ply’*)593 strains and *ply*^*+*^*lytA*^*+*^ of *S*. *pneumoniae*. TNFα in the supernatant was quantified by ELISA (A). Cell viability was measured by lactate dehydrogenase cytotoxicity assay (B). IL-1β production in the supernatant at 24 h with MOI 1 was quantified by ELISA (C). A, B & C Individual data depicted with the mean and SEM from three independent experiments, nd = not detected (limit of detection 29 pg/ml). Data analysed with one-way ANOVA and Dunnett’s multiple comparison post-test with adjusted P values less than 0.05 reported.
(TIF)

S5 Fig Δ(*lytA’-ply’*)593 pneumococci induce less inflammatory cytokines in the serum of mice during an acute pneumonia model than A66 pneumococci. Cytokines from serum 36 hours after wild type C57Bl/6 mice were intranasally inoculated with: 3–8 x 10*4 CFU/mouse of *ply*^+^*lytA*^+^ strain A66 or 8–14 x 10*4 Δ(*lytA’-ply’*)593 strain 2721 CFU/mouse, measured by Luminex bead array. Each data point is one mouse. Uninfected control, mice inoculated intranasally with 50 μl PBS and humanely euthanased at 6 hours.
(TIF)
