# Supplementary figures and images for: Naturally-occurring serotype 3 Streptococcus pneumoniae strains that lack functional pneumolysin and autolysin have attenuated virulence but induce localized protective immune responses
Source: PLoS One. 2023 Mar 10;18(3):e0282843. doi: 10.1371/journal.pone.0282843 (PMC10004606; doi:10.1371/journal.pone.0282843)

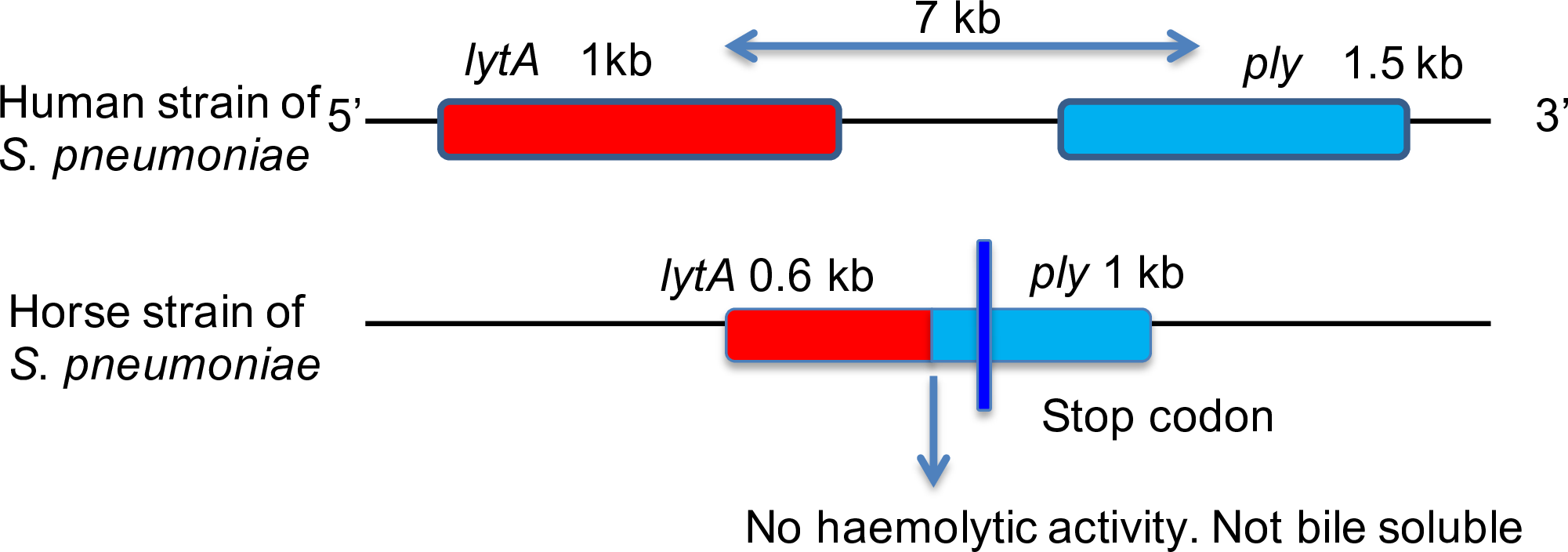

Supplement: S1 Fig — (TIF) [file pone.0282843.s001.tif]

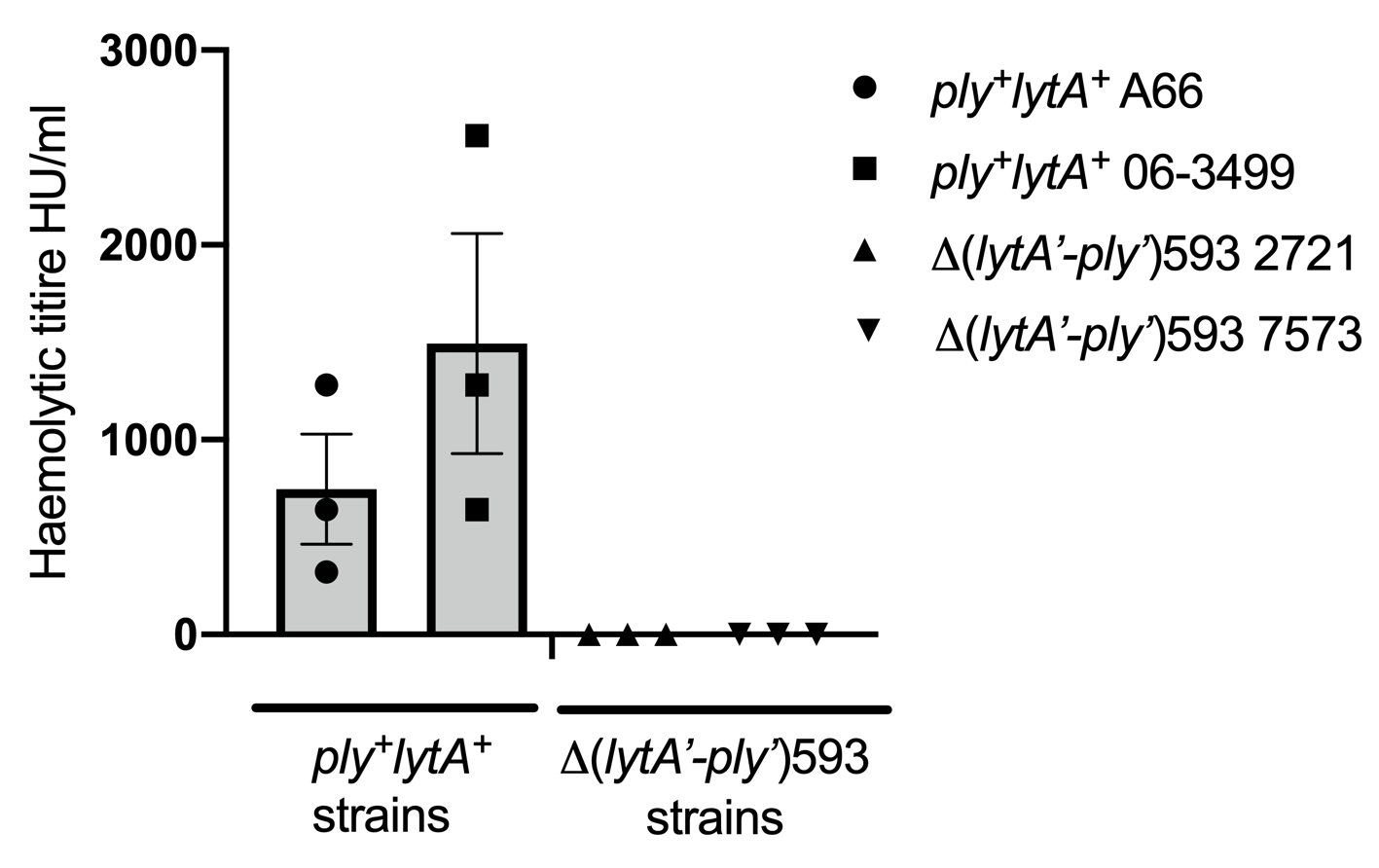

Supplement: S2 Fig — Using a semi-quantitative haemolysis assay, bacterial lysates were serially diluted in the presence of washed equine erythrocytes suspended in PBS and incubated, prior to visual assessment of the degree of erythrocyte lysis. Data depicted are individual data points with the median and range of three independent results, nd = not detected, limit of detection 20 HU/ml. ply+lytA+ strains: A66, 06–3499. Δ(lytA’-ply’)593 strains: 2721, 7573. (TIF) [file pone.0282843.s002.tif]

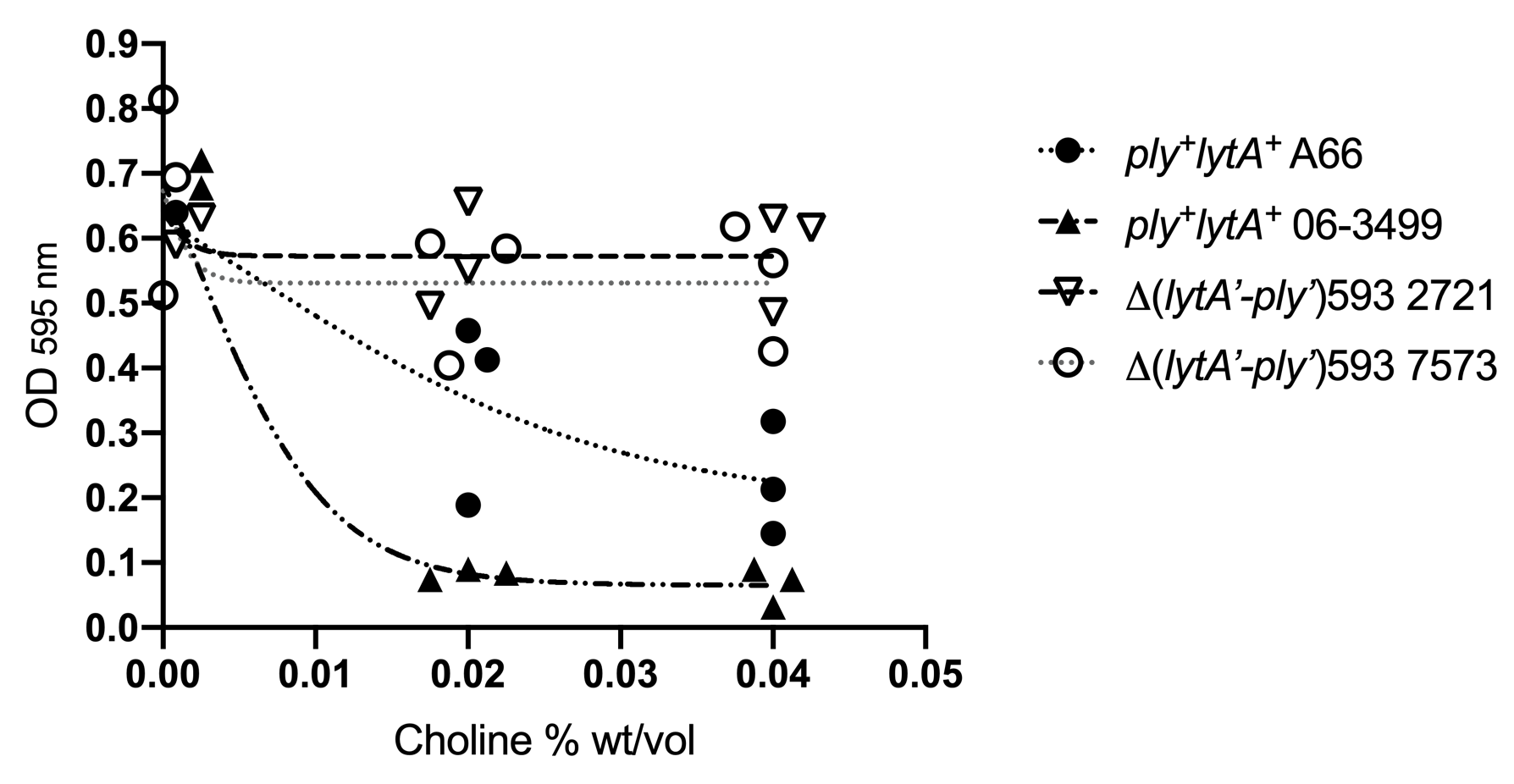

Supplement: S3 Fig — Increasing concentrations of deoxycholate were added to liquid bacterial cultures and the optical density (OD595) of the culture measured after a 5 min incubation. Individual data points depicted of three independent experiments. ply+lytA+ strains: A66 and 06–3499 Δ(lytA’-ply’)593 strains: 2721 and 7573. 0.04% choline data analysed with one-way ANOVA and Dunnett’s multiple comparison post-test with adjusted P values reported. A66 compared to: 06–3499 p = 0.0877, 2721 p = 0.0015, 7573 p = 0.0033. (TIF) [file pone.0282843.s003.tif]

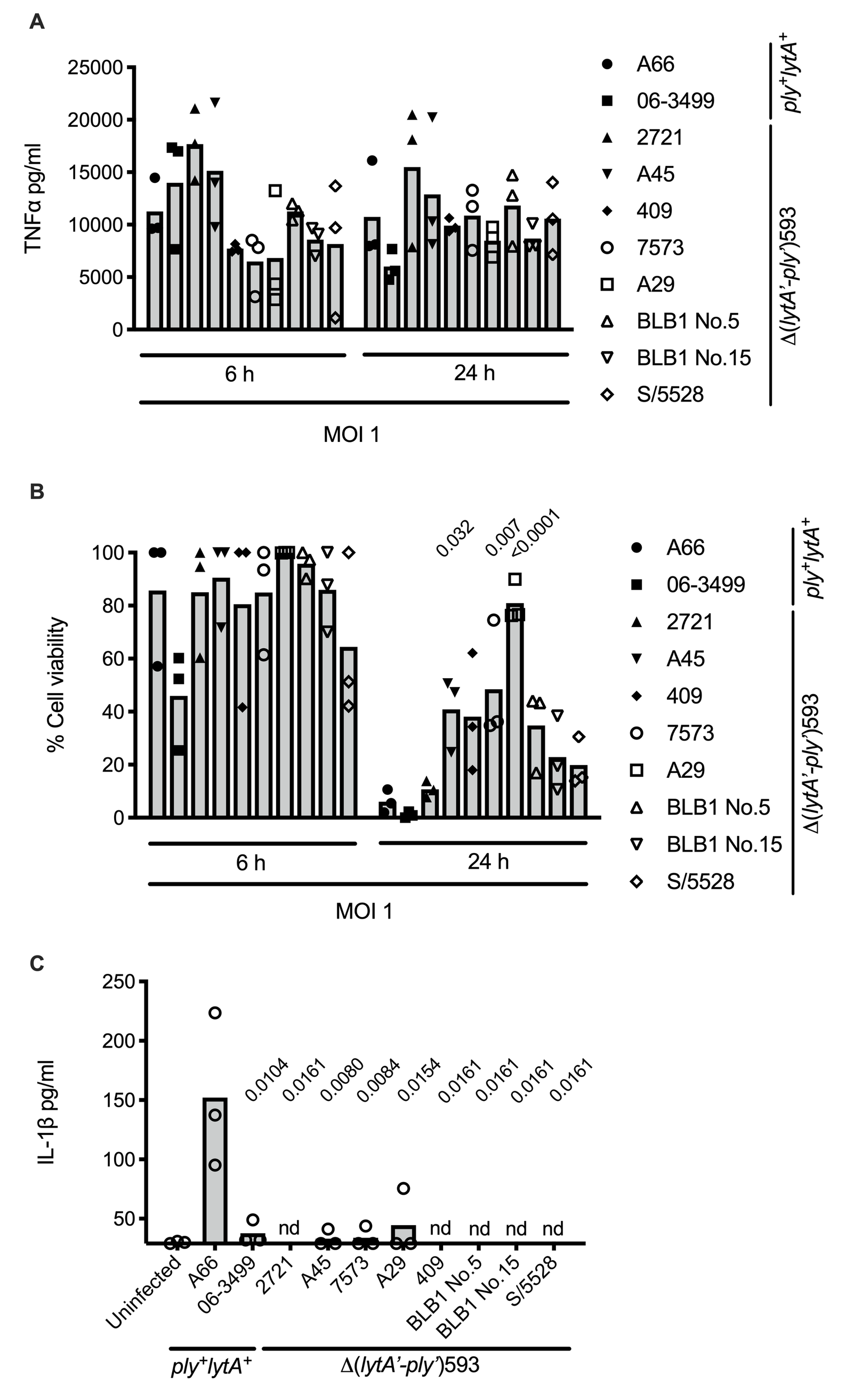

Supplement: S4 Fig — Wild-type murine iBMDM were infected with Δ(lytA’-ply’)593 strains and ply+lytA+ of S. pneumoniae. TNFα in the supernatant was quantified by ELISA (A). Cell viability was measured by lactate dehydrogenase cytotoxicity assay (B). IL-1β production in the supernatant at 24 h with MOI 1 was quantified by ELISA (C). A, B & C Individual data depicted with the mean and SEM from three independent experiments, nd = not detected (limit of detection 29 pg/ml). Data analysed with one-way ANOVA and Dunnett’s multiple comparison post-test with adjusted P values less than 0.05 reported. (TIF) [file pone.0282843.s004.tif]

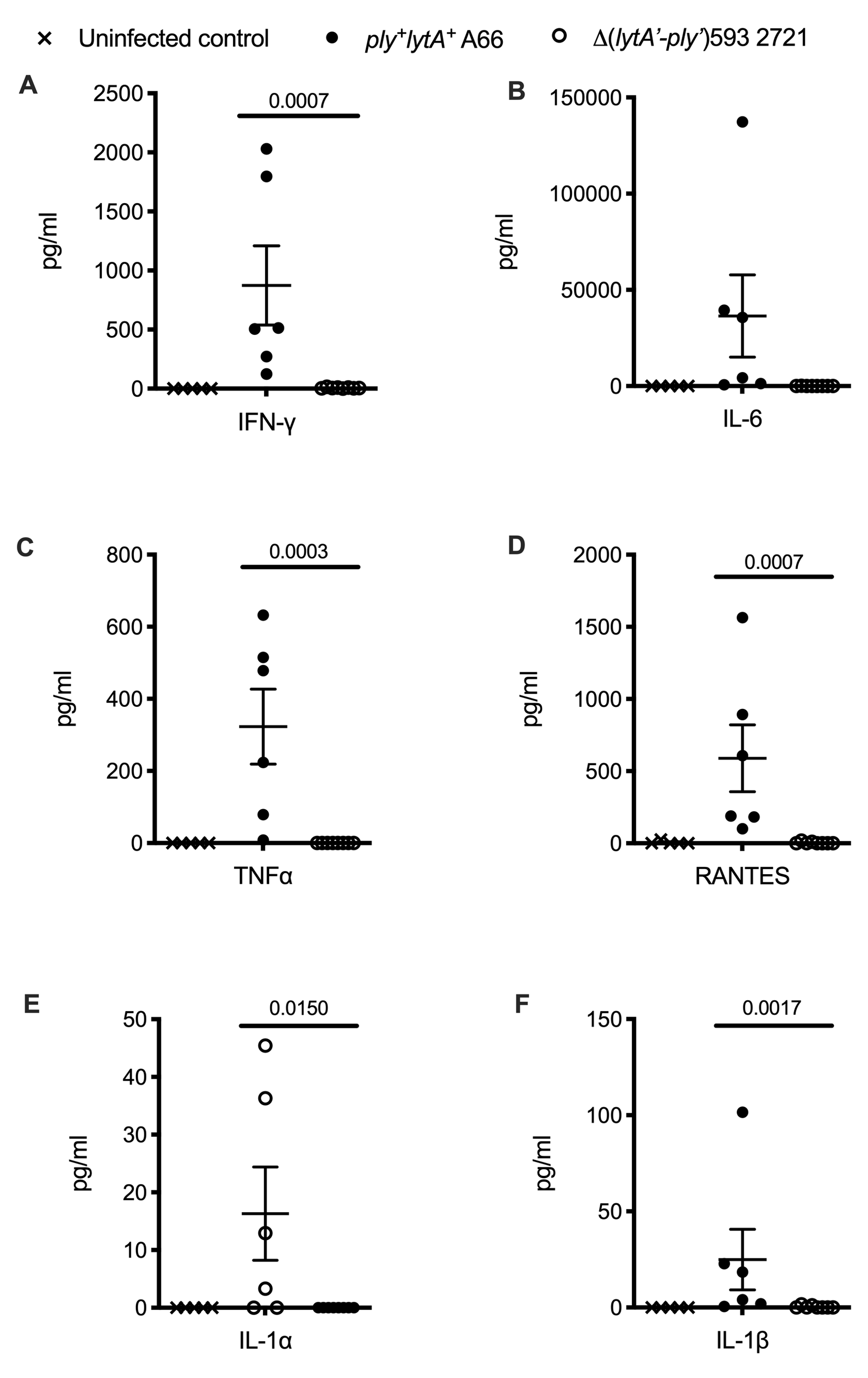

Supplement: S5 Fig — Cytokines from serum 36 hours after wild type C57Bl/6 mice were intranasally inoculated with: 3–8 x 10*4 CFU/mouse of ply+lytA+ strain A66 or 8–14 x 10*4 Δ(lytA’-ply’)593 strain 2721 CFU/mouse, measured by Luminex bead array. Each data point is one mouse. Uninfected control, mice inoculated intranasally with 50 μl PBS and humanely euthanased at 6 hours. (TIF) [file pone.0282843.s005.tif]
